# The Role of Plant Extracts in Enhancing Nutrition and Health for Dogs and Cats: Safety, Benefits, and Applications

**DOI:** 10.3390/vetsci11090426

**Published:** 2024-09-12

**Authors:** Xinzi Guo, Yifei Wang, Zhaoxuan Zhu, Lian Li

**Affiliations:** 1 MOE Joint International Research Laboratory of Animal Health and Food Safety, College of Veterinary Medicine, Nanjing Agricultural University, No. 1 Weigang, Nanjing 210095, China; 15121225@stu.njau.edu.cn (X.G.); zhaoxuanzhu@njau.edu.cn (Z.Z.); 2 College of Animal Science, Nanjing Agricultural University, No. 1 Weigang, Nanjing 210095, China; wangyifei1737@njau.edu.cn; 3 Key Laboratory of Animal Bacteriology, Ministry of Agriculture, College of Veterinary Medicine, Nanjing Agricultural University, No. 1 Weigang, Nanjing 210095, China

**Keywords:** plant extract, antioxidant, dogs, cats, nutrition, diet, supplement, health, gut microbiome

## Abstract

**Simple Summary:**

Companion animals, like dogs and cats, are vital to modern families, and their health is greatly influenced by their diet. Plant extracts, including polyphenols and enzymes, are increasingly used in pet food for their health benefits, such as improving metabolism and gut health. Pet owners are now adding supplements like tea polyphenols, quercetin, and curcumin to their pets’ diets. These natural extracts can replace harmful synthetic antioxidants, extending the shelf life and quality of pet food. Additionally, medical plants offer therapeutic benefits and can help prevent diseases, potentially reducing drug resistance. However, it is crucial to ensure the safety and proper metabolism of these extracts in pets. This review highlights the health benefits of plant extracts in pet diets, aiming to improve dietary practices and overall well-being of companion animals.

**Abstract:**

Plant extracts, derived from various natural sources, encompass primary and secondary metabolites, which include plant polysaccharides, polyphenols, alkaloids, flavonoids, glycosides, terpenes, and volatile oils. These compounds exhibit a range of biological activities such as antioxidant, anti-inflammatory, and antimicrobial functions. Currently, polyphenols and other bioactive compounds are being incorporated into the diets of farm animals, fish, and pets to promote health benefits. Despite this, the application and potential of plant extracts in canine and feline nutrition have not been comprehensively explored. Many aspects of the mechanisms underlying the action of these plant metabolites remain to be analyzed and elucidated. Furthermore, leveraging natural plant extracts for the treatment of clinical conditions in dogs and cats is a crucial component of clinical nutrition. Consequently, this review aims to highlight the impact of plant extracts on overall health, gastrointestinal health, immune health, cardiovascular health, redox balance, and pathology in dogs and cats.

## 1. Introduction

Companion animals play an essential role in modern families, and their overall health is significantly influenced by the supplements and additives extracted from plants in pet food [1]. Plant extracts encompass a wide range of bioactive compounds, including plant polysaccharides, polyphenols, alkaloids, lipids, and enzymes. Polyphenols, in particular, can be categorized into several types, such as phenolic acids, flavonoids, lignins, stilbenes, etc. [2].

In recent years, pet owners have become increasingly aware of the relationship between nutrition and health, leading them to incorporate nutritional supplements such as tea polyphenols, quercetin, and curcumin into their pets’ diets. These functional ingredients have been shown to improve various aspects of health, including metabolism and gut microbiome composition [3].

Moreover, plant extracts offer solutions to challenges associated with pet food storage and the toxicity of synthetic antioxidants like ethoxyquin and sulfites. As natural antioxidants, plant extracts can reduce protein oxidation and lipid peroxidation, thereby extending the shelf life and enhancing the storage quality of pet food [1,4].

In particular, medical plants, containing numerous biologically active substances, can also serve as adjunctive therapies in disease treatment [5]. Furthermore, these natural bioactive substances may play a preventive role in maintaining the health of dogs and cats as feed additives or feed ingredients, thereby potentially reducing the likelihood of developing drug resistance [6,7,8,9,10,11].

Despite these benefits, it is crucial to thoroughly evaluate the metabolism and safety of natural plant extracts in vivo to conduct a comprehensive evaluation. For instance, quercetin has dual effects, inhibiting the digestion but promoting the absorption of proteins in the digestive tract [12,13]. Certain plant extracts may exhibit dose-dependent effects, and careful assessment is necessary to ensure their safe use [14]. Therefore, further empirical studies are required to validate the safety and efficacy of these herbal supplements in pet diets, as standardization and consistent product quality remain critical challenges [15].

This review highlights the metabolism and toxicology of some plant extracts in dogs and cats and the potential nutritional and health benefits of incorporating plant extract as an essential part of supplements in pet diets [16]. Specifically, this review discusses the application of plant extracts in dog and cat diets and their effects on overall health, gut health, cardiovascular health, and redox balance and also how the plant extracts modulate pet pathology to improve their health, aiming to provide valuable insights for dietary nutrition management and adjunctive therapy in dogs and cats [16].

## 2. Metabolism and Toxicology of Some Plant Extracts

The safety of the metabolism of natural plant extracts in dogs and cats depends on various factors, including the type of extract, dosage, and extraction method, as well as the health status and individual variability of the animals [17]. Generally, while many common plant extracts are metabolically safe for dogs and cats, some may pose toxicity risks. Specifically, the appropriate dosage must be tailored based on the specific extract, the animal’s body weight, and its proportion in the daily diet [18,19].

### 2.1. Flavonoids

#### 2.1.1. Flavanols

Tea polyphenols are indeed extracted from tea leaves. These polyphenolic compounds, which include catechins, flavonoids, and anthocyanins, are naturally present in tea leaves. Tea polyphenols exhibit a variety of biological activities, such as antioxidant, anti-inflammatory, antibacterial, and antiviral properties, offering multiple health benefits [20]. Mata-Bilbao et al. (2008) studied the pharmacokinetics of green tea catechins in beagles and found that after oral administration (12.35 mg/kg body weight), catechins such as epigallocatechin gallate (EGCG) and epicatechin gallate (ECG) reached peak plasma concentrations around 1 h. The study reported that conjugated metabolites, particularly EGC-glucuronide, exhibited high area under the curve (AUC) and mean residence time (MRT), indicating prolonged retention due to enterohepatic cycling. However, this study did not report significant toxicity, suggesting that green tea catechins are well tolerated in beagles at the administered doses [21]. However, Kapetanovic et al. (2009) demonstrated that the metabolism of green tea polyphenols, primarily EGCG, in dogs varies significantly between fasted and non-fasted states. In fasted dogs, higher bioavailability leads to increased toxicity, resulting in severe adverse effects including gastrointestinal irritation, weight loss, and mortality. In contrast, non-fasted dogs exhibit lower and less variable exposure levels, leading to reduced toxicity, which underscores the importance of considering feeding status in dosing regimens to minimize the risk of toxicity in clinical applications of green tea polyphenols [17].

#### 2.1.2. Flavanones

Naringin is a flavonoid compound and is classified as a polyphenol. It is widely found in the peel and pulp of citrus fruits, particularly in grapefruit and other citrus species. Naringin exhibits various biological activities, including antioxidant, anti-inflammatory, anticancer, and cardiovascular protective effects. Due to these properties, naringin is extensively studied and utilized in the fields of food, medicine, and dietary supplements. Liu et al. (2012) investigated naringin metabolism in dogs, identifying 22 naringin-related metabolites. Approximately 60% of naringin was excreted, primarily as 4-hydroxyphenylpropionic acid, indicating significant biotransformation [22]. And Li et al. (2020) also revealed that naringin has a good safety profile in beagle dogs, which concluded that the LD50 dosage level was greater than 5 g/kg body weight and the NOAEL was at least 500 mg/kg body weight per day when given orally for 3 and 6 consecutive months [23]. The results provide a foundation for further pharmacological and toxicological studies. As for naringenin, the aglycone form of naringin, it typically exhibits higher bioavailability and directly participates in various biological activities. Specifically, after oral administration, naringin is hydrolyzed by intestinal enzymes and microbiota into naringenin [24]. Naringenin is then absorbed into the bloodstream and undergoes phase I and phase II metabolism in the liver. Phase I reactions include hydroxylation and reduction, while phase II involves glucuronidation and sulfation. These metabolic processes enhance the solubility and excretion of naringenin. It is primarily excreted via urine and bile, with a significant portion undergoing enterohepatic recirculation, prolonging its presence in the body [25].

#### 2.1.3. Isoflavones

Soy isoflavones are naturally occurring compounds found in soybeans and other soy products. They belong to a class of phytoestrogens, which are plant-derived compounds with estrogen-like activity. Redmon et al. (2016) investigated the metabolism of soy isoflavones in cats compared to other species. The study found that cats showed significantly lower glucuronidation rates of soy isoflavones, such as genistein, daidzein, and equol, in liver microsomes compared to dogs and other species. This reduced glucuronidation capacity in cats suggests that alternative metabolic pathways, including sulfation, may predominate. Additionally, cats excreted higher levels of equol in urine compared to dogs, possibly due to differences in the intestinal microbiome. These findings highlight species-specific differences in soy isoflavone metabolism and the potential impact on health [26].

#### 2.1.4. Flavonols

Quercetin, a naturally occurring flavonoid, serves as the aglycone base for its glycoside derivatives, rutin and isoquercetin. Rutin (quercetin-3-O-rutinoside) and isoquercetin (quercetin-3-O-glucoside) differ from quercetin by the addition of sugar moieties, rutin with a rutinoside and isoquercetin with a glucose unit. These glycoside forms enhance the bioavailability and stability of quercetin, while retaining similar antioxidant and anti-inflammatory properties, making them valuable in therapeutic applications [27]. In the body, isoquercetin can be hydrolyzed by enzymes to release quercetin [28]. Reinboth et al. (2010) investigated the bioavailability of quercetin from different glycosides in dogs, administering 30 μmol/kg body weight of quercetin, isoquercetin, and rutin to beagle dogs. The absolute bioavailability of quercetin was about 4%. Isoquercetin exhibited significantly higher bioavailability compared to quercetin aglycone, primarily absorbed in the small intestine. The absorption of quercetin from rutin was delayed but not less effective than quercetin, suggesting that rutin could be a good quercetin source in dogs. However, the potential in vivo effects of quercetin outside the gastrointestinal tract are constrained by its relatively rapid metabolism and relatively low bioavailability [29,30].

### 2.2. Phenolic Acids

#### Hydroxycinnamic Acids

Chlorogenic acid (ChA) is a natural phenolic compound found in plants like coffee beans and blueberries. It primarily exists as an ester of caffeic acid and quinic acid. ChA exhibits antioxidant, anti-inflammatory, antiviral, and antibacterial properties and is widely used in food, health supplements, and pharmaceuticals for its health benefits [31]. After oral administration, ChA showed high biological transformation, with minimal amounts of the parent compound detected in urine and feces. Pharmacokinetic parameters indicated that ChA had a rapid absorption and elimination profile, with a half-life of approximately 1 h. These results underscore ChA’s extensive metabolism in beagles [32].

### 2.3. Plant Extract Mixture

Nowadays, some studies use plant extract mixtures to test its safety for pets; for instance, Martineau et al. (2016) conducted a study to evaluate the renal and hepatic safety of a polyphenol-rich extract from grape and blueberry (PEGB) in dogs. This study involved 24 beagle dogs divided into 4 groups, receiving different doses of PEGB (0, 4, 20, or 40 mg/kg body weight per day) for 24 weeks. Plasma and urine samples were collected to measure routine markers of renal and liver damage as well as specific early biomarkers of renal damage, including cystatin C (CysC), clusterin (Clu), and neutrophil gelatinase-associated lipocalin (NGAL). The results showed that PEGB-specific polyphenols and their metabolites were detected in dog plasma, but there was no evidence of renal or hepatic damage across all measured biomarkers. The study concluded that long-term consumption of PEGB at the tested doses was safe for dogs, with no renal or hepatic toxicity observed [33].

Currently, the metabolism and safety of certain plant extract monomers, aglycones, and mixed forms have been validated in dogs and cats. However, further experiments are necessary to determine the optimal doses for their safe use as food additives or dietary supplements. For mixed plant extracts, pharmacokinetic studies are particularly important, as the metabolic pathways of different substances may overlap or potential chemical reactions may occur between the various extracts.

## 3. Effects of Plant Extracts on Pet Physiology

### 3.1. Overall Health

The overall health of pets can be indicated by macro changes such as weight, skin, coat condition, and metabolism, etc. Campigotto et al. (2020) conducted a study on the use of curcumin as an antioxidant in dog food, revealing no significant differences in weight gain between dogs fed with and without curcumin [4]. Similarly, beagle dogs fed with synthetic antioxidants and a blend of essential oils (clove, rosemary, and oregano) and vitamin E in pet food showed no significant difference in all experimental stages; nevertheless, over time, BW of both groups decreased [34].

As stated by Rees et al. (2001), this study investigated the effects of flaxseed (FLX) and sunflower seed (SUN) supplementation on the skin and hair coat condition of 18 dogs over one month. Both groups showed temporary improvement in hair coat quality, but this was not sustained beyond 28 days. The improvements in skin condition scores were noted around day 14, suggesting that short-term supplementation with FLX or SUN can improve skin and hair coat conditions in dogs due to increased serum 18 carbon PUFA levels [35].

Another study explored the effects of black ginseng (BG) on beagle dogs, and serum samples were analyzed using high-resolution magic angle spinning (HR-MAS) nuclear magnetic resonance (NMR) spectroscopy. Dogs were fed BG tablets for eight weeks, and their blood samples were collected at 0, 4, and 8 weeks. Metabolic profiling revealed significant changes in several metabolites, including increased glucose, BCAAs (isoleucine, leucine, and valine), alanine, glutamine, and histidine, and decreased lactic acid and formate, which indicated that BG administration enhances immunity and energy metabolism, confirming its biological efficacy and potential application in pet health care [36].

The last study investigated the anti-inflammatory effects of BG extracts through serum metabolic profiling in beagle dogs. Dogs were divided into three groups: regular diet (control), medium concentration BG extract (BG-M, 400 mg/10 kg/day), and high concentration BG extract (BG-H, 800 mg/10 kg/day). After eight weeks, significant changes in serum metabolites were observed. The BG-H group showed increased levels of glycine and β-alanine, which demonstrated the potential anti-inflammatory effects of BG extract in beagles. In the author`s study, the AUC values of glycine and β-alanine reached 1.0, demonstrating their strong potential as reliable biomarkers for predicting high concentrations of BG extract intake [37].

### 3.2. Gastrointestinal Health

The digestive capacity of the gastrointestinal tract is significantly influenced by the gut microbiota, and plant extracts have the potential to serve as functional food ingredients [3,38]. Pinna et al. (2017) investigated the impact of *Yucca schidigera* extract (YSE) and chestnut tannins (CTs) on the microbial and metabolic activities in dog and cat feces. Both species showed similar trends, with reductions in alcohols and esters, suggesting a shared response to the antibacterial properties of tannins. However, distinct species-specific differences were observed. In dogs, YSE and CTs primarily influenced cadaverine, ammonia, and sulphur compounds, while in cats, the treatment affected indole and trimethylamine levels. Additionally, CTs exhibited a minor inhibitory effect on *Escherichia coli* in dogs, while YSE increased enterococci populations. These findings highlight the potential for YSE and CTs to beneficially alter gut microbiota and reduce harmful volatile metabolites, though the specific effects vary between dogs and cats, which indicates the potential benefits of incorporating YSE and CTs into pet diets. However, further in vivo studies are required to confirm their efficacy and safety [39].

Barry et al. (2009) studied the effects of low-level fructan supplementation, including inulin and fructooligosaccharides (scFOS), on nutrient digestion and fecal metabolite concentrations in dogs, and they found that both inulin and short-chain scFOS enhanced nutrient digestibility and modified fecal metabolites, such as decreasing phenol concentrations. However, the supplementation did not significantly alter fecal microbiota populations. These findings suggest that while low-level fructan supplementation improves certain nutritional outcomes in dogs, higher inclusion levels may be needed to impact gut microbiota significantly [40].

In addition, Soares et al. (2023) investigated the effects of a blend of yeast cell wall and oregano essential oil (YCO) on nutrient digestibility, diet palatability, and intestinal functionality in dogs. Eighteen adult dogs were fed diets with varying YCO levels (0, 1.5, 3.0 kg/ton) for 20 days, and it was discovered that while the highest YCO level reduced dry matter digestibility, it enhanced fecal bacterial diversity and decreased concentrations of harmful fecal metabolites like ammonia and histamine. This reveals the potential benefits of YCO in improving gut health despite a slight reduction in nutrient digestibility [41].

Additionally, Jewell et al. (2022) evaluated the effects of feeding fiber-bound polyphenol ingredients (pecan shells, flax seed, cranberry, citrus, and beet) to dogs at various concentrations (0%, 1%, 2%, and 4%) over 31 days and found that increasing the fiber-bound polyphenol content in the diet enhanced fecal levels of beneficial bioactive metabolites like short-chain fatty acids (SCFAs) and polyphenols, particularly at 4%. However, there were no significant changes in the fecal microbiota composition. These results suggest that fiber-bound polyphenols can improve gut health by increasing bioactive metabolites and antioxidants in dogs, which is almost the same as in cats [42,43].

### 3.3. Immune Health

Immune balance is crucial for the health of dogs and cats, and plant extract plays an irreplaceable role in modulating immune function [44,45]. Woode et al. (2015) investigated the effects of resveratrol on canine immune function in vitro, and they uncovered that resveratrol was sparing to phagocytosis but significantly reduced the oxidative burst capacity of poly-morphonuclear cells (PMNs) [46].

As conducted by Campigotto et al. (2020), curcumin supplementation resulted in an increase in total erythrocytes, hematocrit, and hemoglobin levels compared to the control group, indicating enhanced red blood cell production and oxygen-carrying capacity. Additionally, there was a significant rise in the total leukocyte count, primarily driven by increased neutrophil and monocyte counts. Conversely, a notable decrease in lymphocytes was recorded in the curcumin-supplemented group, suggesting a modulatory effect on the immune cell distribution, which highlights curcumin’s potential benefits in enhancing certain hematological parameters while modulating immune cell populations in dogs; however, significant increases were observed in glucose, cholesterol, triglycerides, and urea in the supplemented group generally, which may be related to various factors, such as the duration of the experiment, the age of the dogs, and other potential variables. Conversely, total protein, globulin, and alanine aminotransferase (ALT) levels decreased notably. Overall, curcumin was found to enhance animal health, primarily by stimulating the antioxidant system and demonstrating clear anti-inflammatory effects [4].

Sgorlon et al. (2016) conducted a dietary intervention study to investigate the nutrigenomic activity of plant-derived compounds in dogs. They administered four nutraceuticals—*Echinacea angustifolia*, *Vaccinium myrtillus*, *Curcuma longa*, and *Sylibum marianum*—to different groups of dogs over 60 days. The study found that these compounds modulated gene expression and improved various health markers. For instance, the *Vaccinium myrtillus* group and *Curcuma longa* group significantly downregulated pro-inflammatory genes such as *TNF*, *CXCL8*, *NFKB1*, and *PTGS2*, along with a decrease in plasma ceruloplasmin (CuCp), while the *Curcuma longa* group increased the Zn level, which is the same as the *Echinacea angustifolia* group. *Sylibum marianum* showed a decrease in plasma ALT/GPT activity and an increase in paraoxonase, demonstrating that nutraceuticals can beneficially influence immune response and overall health in dogs [47].

Another study was conducted to evaluate the effects of oral administration of *Echinacea* hydroethanolic extract on the immune system of dogs. Fourteen dogs were randomly assigned to two groups: one receiving 1 mL of 5% *Echinacea* extract twice daily for two months and the other receiving a placebo. Hematology and immunology tests were performed on days 0, 30, and 60. Results indicated significant increases in packed cell volume (PCV), hemoglobin (Hb), red and white blood cell counts, lymphocytes, neutrophils, phagocytosis percentage, and IgM levels in the *Echinacea* group, suggesting potential immunostimulatory effects of *Echinacea* [48].

Ginseng total saponin (GTS) has been studied for its immunomodulatory effects on canine peripheral blood phagocytes. The study found that GTS did not directly affect the phagocytic capacity of peripheral blood mononuclear cells (PBMCs) and PMNs but enhanced the phagocytic capacity of PMNs and monocytes through culture supernatant from GTS-treated PBMCs. This enhancement was mediated by TNF-α released from the stimulated PBMCs. Thus, GTS demonstrates an immune-enhancing effect on canine peripheral blood phagocytes, primarily through TNF-α mediation [49].

A study aimed to evaluate the effects of *Echinacea* hydroethanolic extract on the immune system of dogs. Fourteen dogs were divided into two groups: one received 1 mL of 5% *Echinacea* extract twice daily for two months, while the other received a placebo. Blood samples were taken on days 0, 30, and 60 to measure various hematological parameters and the results demonstrated that the group treated with *Echinacea* exhibited significant increases in PCV, Hb, red blood cell (RBC) count, white blood cell (WBC) count, the percent of phagocytosis, and IgM levels, indicating that these extracts might have appreciable immunostimulatory activity [50].

### 3.4. Cardiovascular Health

Generally, vascular endothelial dysfunction precedes cardiovascular dysfunction, and under normal conditions, there is a balance between the proliferation and apoptosis of endothelial cells. Baumgartner-Parzer et al. (2012) explored the effects of pomegranate extract and soy isoflavones on canine aortic endothelial cells (CnAoECs) to address the increasing rate of cardiovascular disease in dogs. The study developed an in vitro model to evaluate these substances’ impact on cell proliferation and apoptosis, which showed that pomegranate extract and soy isoflavones significantly reduced cell proliferation and apoptosis. This indicates their strong vasoprotective activities, suggesting potential benefits for managing canine cardiovascular diseases [51].

### 3.5. Redox Balance

Maintaining redox balance within the animal body is crucial for normal physiological functions, as oxidative stress can lead to cellular damage and may promote aging and the development of various diseases. Given that the lifespan of pets is significantly shorter than that of humans, reducing oxidative stress in their bodies to extend the time they can accompany humans is of great significance [52]. In addition, food oxidation leads to spoilage, affecting its nutritional value, and long-term consumption may pose health risks [53,54].

In a study examining the effects of using curcumin as a substitute for synthetic antioxidants in dog food, curcumin showed good antioxidant effects, presenting that catalase (CAT) activity, superoxide dismutase (SOD) activity, glutathione peroxidase (GPx) activity, antioxidant capacity against peroxyl radicals (ACAP), protein sulfhydryls (PSHs), and non-protein sulfhydryls (NPSHs) significantly increased, reactive oxygen species (ROS) levels greatly decreased, and glutathione s-transferase (GST) did not show specific changes. Also, it demonstrated that curcumin supplementation effectively prolonged the shelf life of dog food by reducing oxidative degradation [4].

As demonstrated by Bang et al. (2022), the supplementation of quercetin at a concentration of 50 μM significantly reduced ROS level and apoptosis index while enhancing motility, survival rates, and mucus penetration in post-thaw dog sperm. These findings indicate that quercetin, at the optimal concentration of 50 μM, is effective in mitigating cryo-induced oxidative stress and improving the quality of post-thaw dog sperm [55].

The study by Schlieck et al. (2021) examines the replacement of synthetic antioxidants with a blend of essential oils (clove, rosemary, and oregano) and vitamin E in dog food, focusing on food quality and health effects in beagles. Ten adult beagles were divided into control and test groups, receiving food with synthetic and natural anti-oxidants, respectively. Results showed natural antioxidants in dog food promote feed conservation, and the natural antioxidant group exhibited a reduced ROS level and increased antioxidant enzyme activity; thus, based on the above information, the natural antioxidants are effective in preserving dog food and enhancing the systemic antioxidant response of dogs [34].

### 3.6. Other Aspects

In addition to the aforementioned effects, natural plant extracts have significant impacts on various other aspects of pet physiology, including cognition and other related functions. This study investigated the effects of a nutraceutical supplement containing phosphatidylserine, *Ginkgo biloba*, vitamin E, and pyridoxine on cognitive function in aged beagles. Nine aged beagles were tested on a delayed-non-matching-to-position task to assess short-term visuospatial memory. The study used a crossover design with the supplement administered in one phase and a control in the other. The results showed that the supplement significantly improved memory performance in the beagles compared to the control, and these effects were long-lasting, which implies the potential of the supplement to enhance cognitive function in aged dogs [56]. For detailed information regarding the relevant summary, please refer to Table 1.

## 4. Effects of Plant Extracts on Pet Pathology

### 4.1. Poisoning Disease

Heavy metal poisoning in dogs and cats is relatively uncommon. However, toxicity from ingesting plants and other substances, such as xylitol, is more frequently observed [57]. Yu et al. (2013) conducted a study examining the protective effects of resveratrol on arsenic trioxide-induced nephrotoxicity in cats, which discovered that resveratrol (3 mg/kg) significantly mitigated oxidative stress and renal damage caused by arsenic trioxide. It achieved this by enhancing the activities of antioxidant enzymes such as glutathione peroxidase (GSH-Px), catalase (CAT), and superoxide dismutase (SOD), reducing malondialdehyde (MDA), reactive oxygen species (ROS), 8-Hydroxy-2′-deoxyguanosine (8-OHdG), creatinine (CREA), and blood urea nitrogen (BUN) levels, thereby inhibiting As_2_O_3_-induced oxidative damage and significantly reducing arsenic accumulation in renal tissues by promoting As_2_O_3_ metabolism [58].

### 4.2. Nutritional Metabolic Disease

Nutritional metabolic disorders are relatively common in dogs and cats, particularly given that modern pets often spend more time indoors than outdoors, coupled with issues related to insufficient physical activity [59]. Obesity is a chronic condition caused by excessive or abnormal accumulation of body fat. It typically results from an imbalance where energy intake exceeds energy expenditure. This condition involves multiple metabolic processes and is closely associated with other metabolic diseases such as diabetes, hypertension, and cardiovascular diseases, linked to inflammation [59,60,61,62]. The development of obesity is influenced by various factors, including genetics, diet, lifestyle, and environmental factors [63]. Managing and preventing obesity generally requires a comprehensive approach that considers dietary, exercise, and lifestyle modifications. Consequently, the regulatory role of plant extracts in managing nutritional metabolic disorders warrants attention.

Based on Kobayashi et al. (2020), the effects of quercetin derivative on healthy and obese cats were evaluated over 4 weeks. Both healthy and obese cats exhibited significant reductions in plasma NEFA and SAA concentrations, as well as in LDH and ALT activities after supplementation. And obese cats experienced significant decreases in TC concentrations and AST and ALT activities. These findings suggest that while the quercetin derivative effectively reduces oxidative stress and inflammation in both healthy and obese cats, the metabolic improvements are more pronounced in obese cats, highlighting the compound’s potential benefits for managing obesity-related conditions [64].

Moreover, according to Li et al. (2020), green tea polyphenols (GTPs) were found to decrease the relative abundance of Bacteroidetes and *Fusobacteria*, while increasing the relative abundance of Firmicutes. The proportions of *Acidaminococcus*, *Anaerobiospirillum*, *Anaerovibrio*, *Bacteroides*, *Blautia*, *Catenibactetium*, *Citrobacter*, *Clostridium*, *Collinsella*, and *Escherichia* were significantly correlated with GTPs-induced weight loss. In addition, GTPs significantly reduced the expression levels of pro-inflammatory cytokines such as TNF-α, IL-6, and IL-1β and inhibited the activation of the toll like receptor 4 (TLR4) signaling pathway compared with a high-fat diet, indicating that the beneficial effects of GTPs are linked to alterations in gut microbiota and reduced intestinal inflammation, which may underlie the anti-inflammatory and anti-obesity properties of GTPs [65].

Similarly, as mentioned by Rahman et al. (2020), decreased TNF-α, IL-1β, and IL-6 expressions were shown in dogs fed tea polyphenols and a high-fat diet after 12 weeks, and TPs suppressed COX-2 and iNOS expression levels, diminishing obesity, liver inflammation, liver fat content, and degeneration [66].

Based on Leray et al.’s (2011) study, eight obese cats were fed two different diets supplemented with either citrus polyphenols (hesperidin and naringin) or highly bioavailable curcumin for two 8-week periods, which showed that both supplements lowered plasma acute-phase protein (APP) concentration. Additionally, citrus polyphenols reduced AGP and haptoglobin levels, while curcumin primarily reduced AGP levels. However, there were no significant differences in cytokine mRNA levels (TNF-α, IL-1β, IL-4, IL-5, IL-10, IL-12, IL-18, and transforming growth factor-β) between the two supplements, except for a decrease in IFN-γ and IL-2 mRNA levels at the end of the citrus and curcumin supplementation periods, respectively. These findings suggest that citrus polyphenols and curcumin can improve obesity-related inflammatory states by targeting liver functions and reducing APP concentration [67].

### 4.3. Circulatory System Disease

Many procoagulant and anticoagulant drugs are derived from plant extracts. As blood is a critical component of the circulatory system, the application of plant extracts in the treatment of circulatory system diseases holds significant potential. Davis et al. (2021) investigated the effects of seven days of oral resveratrol on hemodynamic response and acute kidney injury (AKI) in a canine hemorrhagic shock model. Twelve greyhounds were given either resveratrol or a placebo before inducing hemorrhage. Results showed a higher blood volume loss was needed to achieve hypotension in the resveratrol group, but no significant differences in AKI biomarkers or renal injury scores between groups were found. Resveratrol did not increase bleeding risk and improved blood pressure tolerance to severe hemorrhage, but it did not provide renal protective effects [68].

Fu et al. (2013) investigated the effects of hawthorn leaves flavonoids (HLFs) on acute myocardial ischemia/reperfusion injury in anesthetized dogs. They created an ischemia model by ligating the left anterior descending (LAD) artery for 60 min, followed by reperfusion. The study included three groups of dogs: a control group and two treatment groups receiving low (5 mg/kg) and high (10 mg/kg) doses of HLFs. The results showed that HLFs significantly reduced the degree and scope of myocardial ischemia and MPO and inhibited inflammatory cytokines TNF-α and IL-1. Additionally, HLFs enhanced the expression of G protein-coupled receptor kinase 2 (GRK2) and inhibited the expression of nuclear factor κB (NF-κB) in the ischemic myocardium, which conveys that HLFs have protective effects against myocardial ischemia/reperfusion injury, potentially through anti-inflammatory mechanisms and modulation of GRK2 and NF-κB pathways [69].

### 4.4. Digestive System Disease

Gastrointestinal diseases are relatively common in dogs and cats, involving multiple organs such as the liver and intestines [70]. Gogulski et al. (2021) demonstrated that the digestion would not be affected by pure or commercial hepatoprotectant. However, the commercial hepatoprotectant can reduce ALT, AST, and GGT greatly. Importantly, dietary supplementation with commercial hepatoprotectant containing silybin led to reduced activity of serum liver markers and a decrease in liver-specific miRNA concentrations, thereby improving liver function indices. Thus, silybin supplementation can be an effective therapeutic tool for dogs with liver diseases [71,72].

Yimam et al. (2019) examined the effects of botanical composition, UP446, on ligature-induced periodontal disease in beagle dogs. UP446, which primarily consists of bioflavonoids such as baicalin from *Scutellaria baicalensis* and catechins from *Acacia catechu*, was administered to the dogs at concentrations of 0.1% and 0.2% over 12 weeks. The study found that UP446 significantly reduced gingivitis, pocket depth, clinical attachment loss, and gum bleeding compared to a placebo. These findings suggest that UP446 could be used alone or in combination with other oral hygiene preparations to treat periodontal disease in both humans and companion animals [73].

Zhang et al. (2023) investigated the effects of grape seed proanthocyanidin (GSP) on labrador retrievers with mild inflammatory bowel disease (IBD). GSP alleviated intestinal inflammation by enhancing anti-inflammatory gut bacteria, including *Ruminococcaceae*, *Faecalibacterium*, *Ruminococcus_torques_group*, and *Lachnospiraceae_NK4A136_group*, and beneficial bile acids. The study also used fecal microbiota transplantation to confirm that changes in gut microbiota were crucial for GSP’s effectiveness, proposing a polyphenol-based dietary strategy for canine intestinal health improvement [74].

### 4.5. Oxidative Stress

In the lives of dogs and cats, numerous stressors such as sudden environmental changes can induce oxidative stress. Therefore, utilizing plant extracts as antioxidants to alleviate stress-related issues in dogs and cats presents a natural advantage. Generally speaking, oxidative stress in in vitro culture conditions is likely to be greater, and the addition of TBHP also induces a state of oxidative stress. Barden et al. (2008) investigated the potential protective effects of grape seed proanthocyanidin extract (GSE), resveratrol (RES), and their combination (GSE + RES) against oxidative stress-induced damage in cultured canine lens epithelial cells (LECs). The study demonstrated that GSE and GSE + RES significantly reduced the production of reactive oxygen species (ROS) following exposure to tertiary butyl-hydroperoxide (TBHP). GSE was particularly effective in reducing ROS production and inhibiting stress-induced cell signaling pathways, such as the mitogen-activated protein kinase (MAPK) and phosphoinositide-3 kinase (PI3K) pathways. These findings suggest that GSE has a strong potential to protect LECs from oxidative stress, linked to cataractogenesis, indicating its possible use as a dietary supplement to prevent or delay cataract formation in dogs [75].

In a study examining the effects of *Arctium lappa* (burdock) extract on canine dermal fibroblasts, researchers stimulated cells with hydrogen peroxide (H_2_O_2_) to induce oxidative stress. The cells were then treated with burdock extract, and the study assessed cell viability, adhesion, and gene expression profiles using RNA sequencing (RNA-seq). The findings revealed that burdock extract modulates key signaling pathways, including Wnt/β-catenin and chondroitin sulfate biosynthesis, which are crucial for wound healing. Additionally, the extract upregulated the antioxidant enzyme SOD2, indicating potential antioxidant and anti-inflammatory properties of burdock in canine skin cells [76].

Yang et al. (2022) demonstrated that gallnut tannic acid (TA) effectively reduced stress symptoms in beagle dogs. TA supplementation alleviated stress-induced diarrhea and reduced levels of stress hormones such as cortisol, glucocorticoid, and ACTH. It also lowered the expression of the stress marker HSP70. Analysis of fecal samples revealed that TA promoted the growth of beneficial gut bacteria and increased levels of fecal butyrate, a short-chain fatty acid beneficial for gut health. These findings suggest that gallnut TA has significant potential as a prebiotic for preventing and treating stress-related metabolic disorders by targeting gut microbiota and modulating metabolic profiles [77].

Phrueksanan et al. (2014) proved that *Clitoria ternatea* flower petal extract, which contains phenolic compounds, flavonoids, and anthocyanins, in AAPH-induced oxidation of erythrocytes, can effectively protect erythrocytes from free radical-induced hemolysis and oxidative damage by preventing lipid peroxidation and protein carbonyl formation and maintaining glutathione levels. The antioxidative capabilities of *Clitoria ternatea* suggest its potential application in preventing oxidative stress-related damage [78].

In a study by Pomari et al. (2013), the effects of *Arctium lappa* (burdock) root extract on canine dermal fibroblasts were investigated under oxidative stress conditions induced by hydrogen peroxide (H₂O₂). The results demonstrated that burdock extract significantly increased cell viability and adhesion in H₂O₂-treated fibroblasts. Specifically, the extract upregulated key genes involved in antioxidant defense and cell adhesion, such as superoxide dismutase 2 (SOD2) and chondroitin sulfate N-acetylgalactosaminyltransferase 2 (CSGALNACT2). These findings suggest that *Arctium lappa* extract may have protective effects against oxidative stress and could potentially aid in wound-healing processes [79].

### 4.6. Inflammatory Responses or Other Related Conditions

Inflammatory responses are typically not confined to individual organs but manifest across multiple systems [80]. Additionally, other diseases that do not fall under the aforementioned categories are also listed in this section. Corbee et al. (2022) investigated the efficacy of a nutritional supplement containing green-lipped mussel, curcumin, and blackcurrant leaf extract in dogs and cats with osteoarthritis, which showed that the supplement led to improvements in locomotion and behavior in partial dogs and cats, but several non-responders appeared in both species. Overall, the supplement demonstrated partial positive effects, indicating the need for further research with a larger sample size and longer duration [81].

Another study examined the effects of flaxseed oil on gene expression related to inflammation in dogs. Over 21 days, five beagles and five greyhounds were given Melrose^®^ flaxseed oil (100 mL/kg food). Blood samples were taken on days 0, 15, and 22. The study found that flaxseed oil downregulated *HSP90* and *IL1b* gene expression in greyhounds, but not in beagles. Plasma fatty acid levels correlated with gene expression changes, showing a significant negative correlation between *HSP90*, *IL1β*, and fatty acid concentrations in greyhounds, which suggests that breed-specific responses should be considered in canine dietary management [82].

Krajčíková et al. (2023) focus on pathophysiological mechanisms by the addition of fisetin to the cyclosporine treatment protocol, revealing that tear production did not recover after 7 or 14 days and Matrix metalloproteinase-9 (MMP-9) levels declined after 14 days generally. Therefore, the addition of fisetin to cyclosporine treatment for DED did not restore tear fluid production but was benefited by decreasing tear film MMP-9 [83].

In a comparative study, Soltanian et al. (2020) evaluated the effects of silymarin and hydrocortisone in a canine lipopolysaccharide (LPS)-induced sepsis model. Fifteen dogs were divided into three groups: control (LPS only), silymarin, and hydrocortisone. Results showed that silymarin significantly increased red blood cell count, hemoglobin, and hematocrit levels while reducing serum activities of aspartate aminotransferase, alkaline phosphatase, lactate dehydrogenase, creatine kinase-MB, and cardiac troponin I compared to hydrocortisone and control groups, which suggests that silymarin provides better protection against sepsis-induced organ injury and hematological alterations than hydrocortisone [84].

Liu et al. (2014) studied the effects of naringin on beagle dogs with LPS-induced acute lung injury. They found that naringin at a dose of 12.4 mg/kg has significant mucoactive effects in LPS-induced acute lung injury models in beagles by targeting multiple pathways: it alleviates LPS-induced inflammation, pulmonary edema, goblet cell hyperplasia, and mucus hypersecretion while also promoting sputum clearance by regulating sputum properties and enhancing ciliary activity. These findings highlight naringin’s promising prospects in clinical nutrition for managing respiratory conditions, setting the stage for further research into its therapeutic applications and benefits in respiratory health [85].

Luo et al. (2023) evaluated the effects of *Astragalus* polysaccharide (APS) supplementation on castrated beagle dogs and revealed that the high-dose APS group (800 mg/kg) showed reduced weight gain, which is beneficial for neutered animals, given that pets are prone to obesity after sterilization due to factors such as decreased metabolic rate, reduced activity levels, hormonal changes, and improper dietary management [86]. Also, it improved wound healing. Specifically, APS supplementation improved hematological parameters (decreased RBC and HCT, increased MCH and PLT), serum biochemical markers (decreased ALP and ALT), immune response (decreased CRP, IL-1β, and TNF-α; increased IL-10), and antioxidant defense (decreased cortisol and protein carbonyl; increased GSH, SOD, CAT, and Se-GPx) [87].

In a study on the effects of Entelon150^®^ (grape seed extract) on intravascular bovine pericardium implantation in beagle dogs, results showed that Entelon150^®^ significantly reduced calcium content compared to the control group and attenuated chronic inflammation marked by infiltration of fibroblasts and macrophages. Histopathological and molecular analyses confirmed lower inflammation levels and decreased expression of bone morphogenetic protein 2 in the Entelon150^®^ group. These findings suggest that Entelon150^®^ effectively mitigates post-implantation inflammation and calcification, potentially enhancing the longevity of bovine pericardium implants in dogs [88]. For detailed information regarding the relevant summary, please refer to Table 2.

## 5. Conclusions and Future Directions

Plant extracts possess inherent advantages in enhancing pet health, with a decreased probability of inducing drug resistance. Additionally, numerous plant extracts have been shown to possess pharmacokinetic profiles that are generally safe, effectively reducing pet owners’ concerns about the potential side effects associated with these supplements. Consequently, an increasing number of plant extracts are being validated as metabolizable by pets, leading to broader applications in pet food to support overall health. Furthermore, novel combinations of plant extracts continue to be identified, offering enhanced antioxidant properties and additional health benefits.

Moreover, natural plant extracts can not only improve the health of dogs and cats under normal conditions but also will be more elucidated in terms of nutritional pharmacology, thereby promoting the improvement and rapid recovery of dogs and cats in diseased states.

With a deeper understanding of the complex interactions between the plant extracts, gut microbiome, nutrition, immune system function, etc., we will be able to develop more precise, effective, and alternative methods for promoting pet health.

## Figures and Tables

**Table 1:** Studies of plant extracts on pet physiology.

Classification	Plant Extract and Animal Subjects	Influence	Reference
Overall health	curcumin; beagle dog	no significant differences in weight gain	[4]
	flaxseed (FLX) and sunflower seed (SUN); dog	temporary improvement in hair coat quality	[35]
	black ginseng; beagle dog	↑immunity and energy metabolism	[36]
	black ginseng; beagle dog	↑levels of glycine and β-alanine	[37]
Gastrointestinal health	*Yucca schidigera* extract (YSE) and chestnut tannins (CT); canine and feline fecal cultures	modify the composition of the fecal microbiota, influence the pattern of volatile fatty acids, ↓the presence of potentially harmful metabolites	[39]
	inulin and scFOS; adult dog	↑nutrient digestibility and modified fecal metabolites	[40]
	yeast cell wall and oregano essential oil (YCO); adult dog	↓dry matter digestibility and enhanced fecal bacterial diversity, ↓concentrations of harmful fecal metabolites like ammonia and histamine	[41]
	fiber-bound polyphenol ingredients (pecan shells, flax seed, cranberry, citrus, and beet); adult dog	↑fecal levels of beneficial bioactive metabolites	[42]
Immune health	resveratrol; canine peripheral blood phagocytes	was sparing to phagocytosis but significantly reduced the oxidative burst capacity of PMNs, ↑pro-inflammatory cytokine (IL-6) production, ↓anti-inflammatory cytokine (IL-10)	[49]
	curcumin; beagle dog	↑total erythrocytes, hematocrit, and hemoglobin levels, ↑total leukocyte count	[4]
	*Echinacea angustifolia*, *Vaccinium myrtillus*, *Curcuma longa*, and *Sylibum marianum*; dog (healthy, with arthritis, with liver disease)	modulated gene expression and improved various health markers	[47]
	*Echinacea* hydroethanolic extract; 14 dogs that were referred to the veterinary clinic	↑packed cell volume (PCV), hemoglobin (Hb), red and white blood cell counts, lymphocytes, neutrophils, phagocytosis percentage, and IgM levels	[48]
	Ginseng total saponin (GTS); canine peripheral blood phagocytes	↑phagocytic capacity of PMNs and monocytes through culture supernatant from GTS-treated PBMCs	[49]
	*Echinacea* hydroethanolic extract; healthy household dogs	↑PCV, Hb, RBC count, WBC count, the percent of phagocytosis, and IgM levels	[50]
Cardiovascular health	pomegranate extract and soy isoflavones; in vitro model	↓cell proliferation and apoptosis	[51]
Redox balance	curcumin; young beagle dog	↑CAT activity, SOD activity, GPx activity, ACAP, PSHs, NPSHs, ↓ROS levels	[4]
	quercetin; frozen canine sperm	↓reactive oxygen species (ROS) levels and apoptosis index, ↑motility, survival rates, and mucus penetration	[55]
	essential oils (clove, rosemary, and oregano) and vitamin E; adult beagle dog	promote feed conservation, ↓reactive oxygen species (ROS) levels, ↑antioxidant enzyme activity	[34]
Other aspects	phosphatidylserine, *Ginkgo biloba*, vitamin E, and pyridoxine; aged beagles	improved memory performance	[56]

**Table 2:** Studies of plant extracts on pet pathology.

Classification	Plant Extract and Animal Subjects	Influence	Reference
Poisoning disease	resveratrol; Chinese Dragon-Li cat	↑GSH-Px, CAT, and SOD, ↓MDA, ROS, 8-OHdG, CREA, and BUN levels, ↓As_2_O_3_-induced oxidative damage and arsenic accumulation in renal tissues, ↑As_2_O_3_ metabolism	[58]
Nutritional metabolic disease	compounds containing quercetin derivatives as main components; healthy mix breed cat and obesity disease cat	↓TC concentrations, AST and ALT activities	[64]
	green tea polyphenols (GTPs); canines with high-fat-diet-induced obesity	weight loss, ↓TNF-α, IL-6, and IL-1β, ↓the activation of the TLR4 signaling pathway	[65]
	tea polyphenols (TPs); high fat-fed dogs	↓TNF-α, IL-1β, and IL-6 expressions, ↓COX-2 and iNOS expression levels	[66]
	citrus polyphenols (hesperidin and naringin) or highly bioavailable curcumin; obese cat	↓acute-phase protein (APP) concentration, ↓IFN-γ and IL-2 mRNA levels	[67]
Circulatory system disease	resveratrol; greyhound dog	a higher blood volume loss was needed to achieve hypotension in the resveratrol group	[68]
	hawthorn leaves flavonoids (HLFs); male dog with acute myocardial ischemia	↓the degree and scope of myocardial ischemia, MPO, ↓TNF-α and IL-1	[69]
Digestive system disease	silybin; healthy dog and dog with hepatopathies	↓activity of serum liver markers, ↓liver-specific miRNA	[71,72]
	bioflavonoids such as baicalin from *Scutellaria baicalensis* and catechins from *Acacia catechu*; beagle dog	↓gingivitis, pocket depth, clinical attachment loss, and gum bleeding	[73]
	grape seed proanthocyanidin (GSP); labrador retrievers with mild IBD	alleviated intestinal inflammation by enhancing anti-inflammatory gut bacteria, including *Ruminococcaceae*, *Faecalibacterium*, *Ruminococcus_torques_group*, and *Lachnospiraceae_NK4A136_group*, and beneficial bile acids	[74]
Oxidative stress	grape seed proanthocyanidin extract (GSE), resveratrol (RES), and their combination (GSE + RES); canine lens epithelial cells	↓ROS production, ↓stress-induced cell signaling pathways, such as the mitogen-activated protein kinase (MAPK) and phosphoinositide-3 kinase (PI3K) pathways	[75]
	*Arctium lappa* (burdock) extract; canine dermal fibroblasts	modulates key signaling pathways, including Wnt/β-catenin and chondroitin sulfate biosynthesis, which are crucial for wound healing	[79]
	gallnut tannic acid (TA); beagle dog	alleviated stress-induced diarrhea, ↓stress hormones such as cortisol, glucocorticoid, and ACTH, ↓the stress marker HSP70	[77]
	*Clitoria ternatea* flower petal extract; canine erythrocytes	preventing lipid peroxidation, protein carbonyl formation, and maintaining glutathione levels	[78]
	*Arctium lappa* (burdock) root extract; canine dermal fibroblasts	↑key genes involved in antioxidant defense and cell adhesion, such as superoxide dismutase 2 (SOD2) and chondroitin sulfate N-acetylgalactosaminyltransferase 2 (CSGALNACT2)	[79]
	green-lipped mussel, curcumin, and blackcurrant leaf extract; dog and cat with osteoarthritis	↑locomotion and behavior in partial dogs and cats	[81]
Inflammatory responses or other related conditions	flaxseed oil; beagles and greyhounds	↓HSP90 and IL1β gene expression in greyhounds, but not in beagles	[82]
	fisetin; dog with dry eye disease	did not restore tear fluid production but was benefited by decreasing tear film MMP-9	[83]
	silymarin and hydrocortisone; a low-dose canine lipopolysaccharide (LPS)-induced sepsis model	↑red blood cell count, hemoglobin, and hematocrit levels, ↓serum activities of aspartate aminotransferase, alkaline phosphatase, lactate dehydrogenase, creatine kinase-MB, and cardiac troponin I	[84]
	naringin; beagle dog	alleviates LPS-induced inflammation, pulmonary edema, goblet cell hyperplasia, and mucus hypersecretion while also promoting sputum clearance by regulating sputum properties and enhancing ciliary activity	[85]
	*Astragalus* polysaccharide (APS); beagle dog	reduced weight gain and improved wound healing	[87]
	Entelon150^®^ (grape seed extract); male beagle dog	↓calcium content and attenuated chronic inflammation marked by infiltration of fibroblasts and macrophages	[88]

## Data Availability

No new data were created or analyzed in this study. Data sharing is not applicable to this article.
